# Progress in Institutional Work

**Published:** 1909-10-09

**Authors:** 


### The Hospital

A Journal of

The Medical Sciences and Hospital Administration.

New Series. No. 137, Vol. VI. [No. 1209, Vol. XLVII.] SATURDAY, OCTOBER 9, 1909.

### Progress in Institutional Work.

The history of hospitals and charitable institutions generally is one that remains to be written in its entirety. We commend the writing of it to an enthusiastic friend of hospitals who has the historian's insight, the judge's impartiality, the student's love for facts, and a friend's sympathy. For all these gifts are necessary in him who would do common justice to so complex a subject, and give fair due to each division in his treatment of it. There is, perhaps, no more interesting, as there is no more fascinating, subject than the history of the progress and reform in institutional work during the last century in general and during the past three decades in particular. It is a progress that largely synchronises with parallel developments in other charitable and scientific work, but that has also points of its own and independent features which are worth consideration. Nevertheless, although it is well to acknowledge that hospital progress has in such ways been independent, it is only fair to admit that it owes much to the discoveries and developments in other branches of work. The economic aspect, for instance, has been influenced by the general development in economic science, the hygienic by the reforms introduced in large buildings such as barracks, prisons, and manufactories, and the architectonic by the evolution of so-called institutional styles and modifications of them. For that reason hospitals owe a burden of gratitude to men like Howard, whose hospital reform work was secondary only to his prison reform work, to the great German and French hygienists, and to women like Miss Nightingale, whose influence on the question of hospital nursing has been world wide. An acknowledgment of this indebtedness in this country has never been wanting, for men connected with hospital work are among the most unselfish and self-denying to be found in any profession. There is, indeed, a catholicity of interest among them which affords an example to members of the medical profession. Questions of priority and unseemly squabbles about nebulous theories seldom if ever arise in the discussions among institutional workers, who, as a whole, are ever ready to share their discoveries with their colleagues and to distribute evenly the limited honour which their labours bring them.

In reviewing as a whole the trend of hospital progress during the past forty years, there are certain outstanding points that appeal to the imagination at once. First of these is undoubtedly the internal rearrangements in hospital work and its various subdivisions which have been brought about by the introduction of new methods in clinical, pathological and general research work. We are the last to suggest that hospital authorities should ever lose sight of the primary important fact that their institutions exist for the benefit of the sick, not for the elucidation of obscure problems in science; but can it be maintained, by logical reasoners, that some attention to the second will seriously interfere with a due regard to the first? Rather, on the contrary, will that institution benefit its patients, its personnel and itself, when it supplies adequate provision—we do not say excessive provision—for the investigation of such points as demand attention in ordinary everyday clinical work. And it is satisfactory to note that the trend in modern hospital work is towards affording such facilities even in comparatively small institutions. In some, notably in American and some Continental hospitals, the research department almost bids fair to overshadow the purely clinical division. Such institutions are scarcely hospitals in the true sense, and it would be wiser to call them auxiliaries to the research department, as, indeed, will be done in the case of the hospital which is to be attached to the New York Rockefeller Institute. The proper attitude of the hospital worker is to be carefully solicitous for the interests of his sick inmates, without disregarding the justifiable claims of members of the staff who are desirous of making use of hospital material for research work, which, no matter whether it is secondarily selfish and a medium of professional advertisement, is primarily and principally calculated to help humanity in its struggle against the "giant agonies of the world."

Yet another point is the co-operation of hospital constructors with medical men, and with those who have had an intimate experience of hospitals, in the designing and building of new institutions. Formerly the hospital architect drew up the plans and specifications for a new hospital without consulting the medical and surgical, or even the administrative staff. He was the sole judge of what was right and proper, although there were instances in which he had no great experience of hospital building, and no special qualifications for designing such structures. The result was, in the majority of cases, what could have been expected, and there are hospitals extant to-day which can serve as dreadful warnings of such awful "individualism." Nowadays where co-operation has prevailed the architect designs in consultation with men who have had magnificent experience in general hospital work, or with members of the staff who will have to use the buildings, and may, therefore, be expected to have some interest in their proper construction. The result has been the erection of such models of hospital construction as the Glasgow Infirmary, the Virchow Hospital at Berlin, and the new Surgical Clinic at Graz. The advantages of co-operation are not yet sufficiently appreciated by hospital workers. When they are fully recognised there will be a considerable improvement in the architectural and structural features of modern hospitals.

